# Dry Ball-Milled Quinoa Starch as a Pickering Emulsifier: Preparation, Microstructures, Hydrophobic Properties and Emulsifying Properties

**DOI:** 10.3390/foods13030431

**Published:** 2024-01-29

**Authors:** Ying Chen, Xue Han, Dong-Ling Chen, Yi-Ping Ren, Shi-Yu Yang, Yu-Xuan Huang, Jie Yang, Liang Zhang

**Affiliations:** School of Food Science and Engineering, Yangzhou University, Huayang Xilu 196, Yangzhou 225127, China; 008265@yzu.edu.cn (Y.C.); mz120222077@stu.yzu.edu.cn (X.H.); 102331@yzpc.edu.cn (D.-L.C.); dx120210217@stu.yzu.edu.cn (Y.-P.R.); 202404229@stu.yzu.edu.cn (S.-Y.Y.); 202404211@stu.yzu.edu.cn (Y.-X.H.); 008305@yzu.edu.cn (J.Y.)

**Keywords:** ball-milling, quinoa starch, Pickering emulsion, emulsifying property, microstructures, hydrophobic property

## Abstract

This research supplied a “cleaner-production” way to produce “clean-label” quinoa starch-based Pickering emulsifier with excellent emulsifying properties. The effects of dry ball-milling time and speed on the multi-scale structures and emulsifying properties of quinoa starch were studied. With increasing ball-milling time and speed, particle size first decreased and then increased, the crystallinity, lamellar structure and short-range ordered structure gradually decreased, and contact angle gradually increased. The increased contact angle might be related to the increased oil absorption properties and the decreased water content. The emulsification properties of ball-milled quinoa starch (BMQS)-based Pickering emulsions increased with the increase in ball-milling time and speed, and the emulsions of BMQS-4 h, 6 h, 8 h, and 600 r reached the full emulsification state. After 120 days’ storage, the oil droplets of BMQS-2 h (BMQS-400 r) deformed, the oil droplets increased, and the emulsification index decreased. The emulsification index and the oil droplets of BMQS-4 h, 6 h, 8 h and 600 r-based emulsions did not show obvious changes after storage, indicating the good emulsifying stability of these BMQS-based emulsions, which might be because that the relatively larger amount of starch particles that dispersed in the voids among the oil droplets could act as stronger network skeletons for the emulsion gel. This Pickering emulsifier was easily and highly efficiently produced and low-cost, having great potential to be used in the food, cosmetic and pharmaceutical industries.

## 1. Introduction

In recent years, clean-label food emulsifiers are much more favored by consumers. The development of Pickering emulsions stabilized by solid particles can reduce the use of chemical surfactants in food and meet consumers’ need for green and safe emulsifiers. There are many food-grade particles that can be used to stabilize emulsions, such as proteins [1], lipids [2], and polysaccharides [3]. Compared with proteins and lipids, polysaccharides, such as starch and cellulose, are abundant in nature; emulsions based on these are much more resistant to environmental changes, such as pH and ionic strength [4].

Starch, as a food material, is degradable, has low cost, and has absolute security [5]. Starch-based Pickering emulsions have great application prospects in many industries, such as the cosmetics, medicine, papermaking and food industries [6,7]. However, natural starches do not have good emulsifying properties, mainly due to their large size and higher hydrophilicity [8]. They are always modified to improve their emulsifying properties.

There are many methods that can be used to improve the emulsifying properties of starch, such as chemical modification, enzymatic modification, and physical modification. Octenyl succinic acid (OSA) chemical modifications are the most-used method to improve the hydrophobic properties of starch particles and increase their emulsifying properties [9]; however, OSA modified starch is relatively expensive, and this chemical modification method is not clean-label. Acidolysis is another chemical method to improve the emulsifying properties of starch [10]; however, much more time (about one week) is needed for this modification, and the processing is not environmentally friendly due to the sulfuric acid or hydrochloric acid used. Enzymatic modification can also decrease the particle size and increase the hydrophobic properties of starch, thus improving the emulsifying properties of starch [11,12]; however, the regulation of enzymatic reaction is relatively delicate and complex. Moreover, for all these modification methods, after the modification process, the pH-adjusting, washing, centrifugating, drying and grinding processes are needed to obtain the final product, which is also reagent-, time- and energy-consuming. Ball-milling is a physical modification method, which has already been used to prepare starch-based emulsifiers [13,14]. Compared with the other modification methods, ball-milling can finely grind particles, which is a relatively simple, cost-effective, and environmentally friendly method [15]. There are two ball-milling methods: dry ball-milling and wet ball-milling. Dry ball-milling is much preferred, since it is a clean-label method without any added reagents that does not produce any pollutants [16]. For wet ball-milling, it is necessary to add some solvents, such as water [13,14], ethanol [17], or acid-based reagents [18], and after wet milling, postprocessing, such as centrifugating, drying, and grinding, is also needed to obtain the final product. Lu et al. [13,14] used wet ball-milling to modify normal, waxy, and high-amylose corn starch with different concentrations, and the obtained starches were used to stabilize the Pickering emulsions. It was found that the longer the milling time, the stronger the emulsifying ability (emulsifying properties: when forming an oil-in-water emulsion system, the maximum amount of oil that can be emulsified per unit of emulsifier) of starch particles due to the decreased starch particle size [13]. Ball-milled high-amylose maize starch showed the highest emulsifying properties among the three kinds of modified starches (normal maize starch, high-amylose maize starch and waxy maize starch) [13]. The starch concentrations during ball-milling also affect the structures and emulsifying properties of corn starch [13,14]. Nevertheless, the wet-milling (starch concentration: 2.4–10.0% *w*/*w*) method was used in their research. However, the wet-milled starch suspensions (2.4–10% *w*/*w*) were directly used to prepare Pickering emulsions in their research, focusing on their potential for commercial production in the future, due to the transportation and storage costs, and the necessary centrifugating, drying and grinding processes, which are time- and energy-consuming [13,14]. Moreover, wet-milling will affect production efficiency due to the large amount of solvent that was used (90.0–97.6%). Liu et al. [19] prepared dry milling areca taro starch; however, full emulsification was not achieved, and OSA modification was further introduced, in combination with ball-milling, to reach a better emulsification effect, which increased the working procedures and costs. The obtained product was not clean-label.

Native quinoa starch was reported to have a better emulsifying capacity than other small-sized starches, such as rice starch and amaranth starch [20]. The emulsifying properties of native quinoa starch cannot meet the standard for practical application. A total of 2.9% OSA was used to modify quinoa starch, while the emulsification index and stability of the related emulsions could be adjusted by adjusting the modified starch concentration and oil–water ratio [21]. Quinoa starches with different OSA modification degrees and emulsions based on these were prepared; the emulsions based on quinoa starch with the highest modification degree had the highest oil droplet size, and the lowest amount of free starch [22]. A dry heat treatment was also used to modify quinoa starch; however, the modified starch did not have good emulsifying properties [22]. α-amylase and the saccharifying enzyme were used to modify quinoa starch to improve its emulsifying properties. The decreased particle size and increased contact angle were reported to be the main reasons for the improved emulsifying properties; however, no full emulsification state was achieved for this kind of modified quinoa starch, with a starch concentration no higher than 3% (*w*/*w*) [11]. The nanoprecipitation method was used to prepare quinoa starch nanoparticles, it was found that under a pH of 3–9 and ionic strength of 0–200 mM, starch nanoparticles with concentration of 2.0–2.5% showed good emulsifying properties with an oil volume fraction in the range of 0.33–0.67, and the quinoa starch nanoparticle-based emulsions showed good free-thawing stability [23]. However, no research related to ball-mill-modified quinoa starch and an emulsion based on this has been reported.

In this research, dry ball-milling, a cleaner production method, was adopted to modify quinoa starch. This was expected to obtain a kind of starch-based emulsifier which is clean-label, has a high producing efficiency (is easily and quickly prepared), and has excellent emulsifying properties and good stability. In this paper, quinoa starch was treated with different milling times and speeds to prepare different ball-milled quinoa starches (BMQS) as Pickering emulsifiers. The effects of milling time and speed on the particle morphology, particle size distribution, crystalline structure, short-range ordered structure, lamellar structure, contact angle, and oil absorption properties of quinoa starch, and their effects on the emulsifying properties and stability of BMQS-based emulsions were comprehensively investigated. The relationship between the structures, oil-absorption and hydrophobic properties, and the emulsifying properties, were discussed to infer the related emulsifying mechanism. This research supplies a cleaner production and clean-label products, which are easily and efficiently produced (only milled for a few hours, without washing, centrifugating, drying and grinding processes), of low-cost, and have good emulsifying properties, with great potential to be used in the food, cosmetic, and pharmaceutical industries.

## 2. Materials and Methods

### 2.1. Materials

Quinoa seeds and soybean oil were purchased from Haishang International food franchise store and Yihai Kerry Arawana Holdings Co., Ltd. (Beijing, China). Nile Blue A, Nile Red and 1, 2-propylene glycol were purchased from Shanghai Macklin Biochemical Co., Ltd. (Shanghai, China). ProClin™150 formaldehyde was purchased from Shanghai Aladdin Biochemical Technology Co., Ltd. (Shanghai, China). Total protein (TP) assay kit (A045-2-2) was purchased from Nanjing Jiancheng Bioengineering Research Institute (Nanjing, China). All other chemicals were of analytical grade and purchased from Sinopharm Group Chemical Reagent Co., Ltd. (Shanghai, China).

### 2.2. Extraction of Quinoa Starch

Quinoa was soaked in Na_2_S_2_O_5_ solution (0.45%, *w*/*v*) with a solid–liquid ratio of 1:3, refrigerated for 24 h, and stirred every 8 h to allow quinoa to soak fully. The soaked quinoa was mashed with a Waring blender with 1 min pause after working for 1 min until it was fully smashed. The slurry was screened through 80 mesh, 160 mesh, 200 mesh, and 400 mesh in sequence to remove insoluble grain residues. The remaining crude starch slurry was mixed and centrifuged at 4000× *g* for 20 min. After centrifugation, the upper yellow brown impurities were scraped off with a scraper, the crude starch was redispersed in distilled water, and the impurities were repeatedly washed and scraped off 3 times. Then, the crude starch precipitate was dispersed in 0.3% NaOH solution, soaked for 20 min and centrifuged. This alkaline processing was repeated twice. Then, distilled water was used to wash the starch, followed by citric acid (pH 4.5) washing. After each washing, the supernatant was removed by centrifugation at 4000× *g* for 20 min. Finally, the starch was washed with distilled water 3 times, and the purified quinoa starch was obtained by centrifugation. The collected starch was dried in a drying oven at 45 °C for 48 h. After drying, the starch was grinded into fine particles, screened through 200 mesh, packed in a sealed bag, and placed in a silica gel drier for further usage.

### 2.3. Preparation of BMQS

A total of 10 g of extracted quinoa starch was added to the chamber of the ball-milling-machine (F-P2000; Hunan Fucas, Changsha, China); then, 100 g of balls (both large and small balls) were added. In order to prevent starch from sticking to the wall during the milling process, two large spherical balls (with a diameter of 15 mm) were mixed with the small spherical balls (with a diameter of 3 mm), and the direction of rotation was adjusted to be alternately clockwise and counterclockwise. BMQS with different milling times of 2 h, 4 h, 6 h, and 8 h were prepared at the same milling speed of 400 rpm; BMQS with different rotational speeds of 200 rpm, 400 rpm, and 600 rpm were prepared at the same milling time of 2 h. All these milling treatments were carried out continuously without pausing. The temperature of the starch sample in the chamber was measured immediately after the ball-milling treatment; it was found that the temperatures were all no higher than 40 °C. The samples with different ball-milling times and speeds were labeled as BMQS-2 h, BMQS-4 h, BMQS-6 h, BMQS-8 h, BMQS-200 r, BMQS-400 r, and BMQS-600 r, respectively. All the samples were prepared three times.

### 2.4. Characterization of BMQS

#### 2.4.1. Protein Content Measurement

Total protein (TP) assay kit was used to test the protein content of QS and BMQS. A total of 1 mg of starch was added into 1 mL of ultra-pure water, and the mixture was vortexed. A total of 50 μL of the starch dispersion was taken out and put into a tube; 3 mL of Coomas bright blue solution was added to the starch solution. The mixture was blended and stood for 10 min. Then, the mixture was put into a 759S UV-Vis spectrophotometer (Lengguang Technology, Shanghai, China) to test the absorbance value at 595 nm. For the blank sample, 50 μL of the dispersed starch solution was replaced by 50 μL ultra-pure water; for standard control, 50 μL of the dispersed starch solution was replaced by 50 μL of standard product. The following processing was carried out in the same way. The protein content can be calculated using Equation (1), as follows:

(1) $$\mathrm{Protein}\;\mathrm{content}\;(g/L)=\frac{A-A_{blank}}{A_{standard-A_{blank}}}\times C_{standard}\times N$$

where *C_standard_* is 0.524 g/L; *N* is the dilution ratio. *A* is the absorbance of the starch sample, *A_blank_* is the absorbance of the blank sample, and *A_standard_* is the absorbance of the standard sample.

#### 2.4.2. Particle Size Measurement

A total of 1 mg of QS or BMQS was dispersed into 10 mL ultrapure water and homogenized at 12,000 rpm for 2 min. Starch dispersion was immediately added to the colorimetric dish of the Laser particle size tester (Winner-802; Jinan Winner; Jinan, China). The dispersion medium was set as water; the refractive index of the medium was 1.333. The particle size distribution of QS and BMQS was tested at room temperature.

#### 2.4.3. Scanning Electron Microscopy (SEM)

The Zeiss field-emission scanning electron microscope (Gemini SEM 300; Zeiss; Berlin, Germany) was used to observe the morphological structures of samples. The samples were dried in an oven at 45 °C for 3 h before observation. After drying, the starch samples were dispersed on the double-sided tape of the sample stage, followed by gold spraying. The starch samples were observed, and pictures were taken at 5000×, 10,000×, and 20,000×, respectively.

#### 2.4.4. Morphology of Starch Dispersion

A total of 10 mg of quinoa starch was dispersed in 5 mL ultra-pure water; 10 μL of starch dispersion was taken on a microscope slide, with the cover slide covered. A total of 0.02% iodine solution was dropped from one side of the slide to stain the starch particles for 2 min. The sample was observed under a fluorescent inverted optical microscope (XDS-600C; Shanghai Caikang; Shanghai, China).

#### 2.4.5. Fourier Transform Infrared (FTIR) Spectroscopy

An appropriate amount of quinoa starch and KBr were mixed, grinded, and pressed into sheets. Then, an FTIR spectrophotometer (Cary 610/670; Varian; Palo Alto, CA, USA) was used to test the short-range ordered structures of the starch samples. The wavenumber range was set from 4000 cm^−1^ to 400 cm^−1^ for the testing, and the scans were carried out 32 times.

#### 2.4.6. Small Angle X-ray Scattering (SAXS)

The QS and BMQS were measured using a small-angle X-ray scatter (NanoSTAR; Bruker AXS; Karlsruhe, Germany). Before measurements, the quinoa starch sample was mixed with ultra-pure water to prepare the 20% starch dispersion, and the dispersion was stood at room temperature for 12 h to be fully wetted. Before the test, starch dispersion was centrifuged at 8000 rpm for 3 min; then, a pipette was used to remove excess water from the upper layer. The fully wetted precipitate was picked up with an ear scoop and placed into the small holes of the sample plate, and both sides of the holes were sealed with tape. Under the X-ray source of monochrome Cu-Kα rays, λ of 0.154 nm, with a tube pressure of 40 kV and tube flow of 50 mA, the starch samples were tested for 10 min, and the corresponding starch spectra were obtained.

The average repeat distance of lamellar structures for each sample can be calculated according to Formula (2), as follows:

(2) $$d_{\mathrm{bragg}}=\frac{2\pi }{q_{c}}$$

where d_bragg_ is the Bragg layered repeat distance; *q_c_* is the location of peak center.

#### 2.4.7. X-ray Diffractometry (XRD)

The crystalline structure of quinoa starch was analyzed by using an X-ray diffractometry machine (D8 Advance; Bruker AXS; Germany). With Cu-Kα ray as the X-ray source, λ of 0.154 nm, a pipeline voltage of 40 mV, pipeline current of 40 mA, scanning range of 3–40°, and scanning speed of 3°/min, the test was carried out. Before the measurements, the starch sample was planished in the sample cell with a glass slide. After the test, the corresponding XRD spectra was obtained. Then, the relative crystallinity of the starch was calculated by Diffrac Eva software of version 2.1.

#### 2.4.8. ^13^C CP/MAS NMR

The cross-polarization/magic angle rotating angle nuclear magnetic resonance (AVANCE III 400 MHz WB; Bruker; Germany) was used for the test. According to the method of Wang et al. [24], the measured parameters were as follows: rotation speed of 4 kHz, frequency of 100.63 MHz, contact time of 2 ms, cycle delay time of 2 s, and acquisition time of 25.17 ms. The spectra in the region of 40–120 ppm were obtained from the Origin 2021 software.

#### 2.4.9. Contact Angle Measurement

Contact angle was tested by using a video optical-contact-angle measuring instrument (OCA20; Data Physics; Filderstadt, Germany). A tablet press was used to press starch into tablets (thickness of 2 mm; diameter of 10 mm). Starch tablets were completely submerged in soybean oil; the needle was also submerged in soybean oil. Then, a high-precision syringe system was used to drop 2 µL of ultra-pure water onto the surface of the tablet. A high-speed camera mounted on the OCA 20 recorded the shape changes of the droplets at 25 frames per second. The contours of water droplets were automatically fitted to the LaPlace-Young equation with OCA 20 software to determine the contact angle of different starch samples.

#### 2.4.10. Oil and Water Absorption Measurements

A total of 0.5 g of starch was weighed and placed in a centrifuge tube; 5 mL of ultra-pure water (or soybean oil) was added to the tube. The blends were stirred with a magnetic agitator (400 r/min) for 30 min, and centrifuged at 4000 rpm for 20 min. The precipitate was reserved. The water absorption–oil absorption rate of starch was calculated according to Formula (3), as follows:

(3) $$\mathrm{Absorption}\;\mathrm{rate}\;(\%)=\frac{m_{2}-m_{1}}{m_{1}}$$

where *m*_1_ is the original starch mass, g; *m*_2_ is the starch mass after water or oil absorption, g.

### 2.5. Preparation and Measurement of Pickering Emulsion

#### 2.5.1. Preparation of Pickering Emulsions Stabilized by Native QS and BMQS

Pickering emulsion was prepared using QS and BMQS as solid-particle emulsifiers. A total of 0.28 g of starch was added into 3.5 mL of ultrapure water to reach full dispersion. A total of 2 μL of ProClin™ 150 preservative was added into the dispersion, followed by the addition of 3.5 mL of soybean oil. The blends were homogenized at 12,000 rpm for 2 min (1 min on + 1 min off + 1 min on) with a high-speed homogenizer (FSH-2A; Changzhou Yuexin; Changzhou, China).

#### 2.5.2. Visual Appearance and Emulsification Index (EI)

The prepared emulsion was kept at room temperature and photographed for four months. Emulsification Index (EI) was obtained by dividing the emulsion layer height by total height. The EI calculation Formula (4) is displayed below:

(4) $$\mathrm{EI}=\frac{H_{e}}{H_{t}}$$

where *H_e_* is the emulsion layer height (cm), and *H_t_* is the total height (cm).

#### 2.5.3. Optical Microscope Observation

After 1 day, 7 day, 14 days, 21 days, 28 days, 75 days, and 120 days, 10 µL of the above-mentioned Pickering emulsion was taken and placed on the slide, with a cover slide carefully covering the emulsion. The morphology of the Pickering emulsion during storage was then observed by using a fluorescent inverted optical microscope (XDS-600C; Shanghai Caikang; Shanghai, China) at a magnification of 100 times.

#### 2.5.4. Confocal Laser Scanning Microscopy (CLSM) Observation

According to the experimental method of Zhang et al. [25], the distribution of starch particles and oil droplets in the emulsion after storage for 1 days and 28 days was observed using a CLSM machine (LSM 880NLO; Carl Zeiss; Jena, Germany). The Nile Blue A and Nile Red dyes were dissolved in ultra-pure water and 1, 2-propylene glycol, respectively, to prepare 0.1 mg/mL dye solution. The two dye solutions were mixed well according to the volume ratio of 1:1. The above-mentioned dye solutions should be kept away from light. A total of 100 μL of the mixed fluorescent dye solution was added to 1 mL Pickering emulsion, and the dyed emulsions were then observed. Nile Blue A (starch dyes) and Nile Red dyes (oil dyes) were excited by a He-Ne laser at 633 nm and an Ar laser at 488 nm, respectively.

### 2.6. Statistical Analysis

All the above experiments were repeated three times, and the data were expressed as mean ± standard deviation (x ± sd). SPSS 25.0 software was used for significance analysis, the one-way analysis of variance (ANOVA) test was used first, and then Tukey (T) multiple comparison test was used. The significant difference was marked with different letters (*p* < 0.05).

## 3. Results and Discussion

### 3.1. Morphological Structures and Particle Size

#### 3.1.1. Starch Morphology

**Figure 1:**
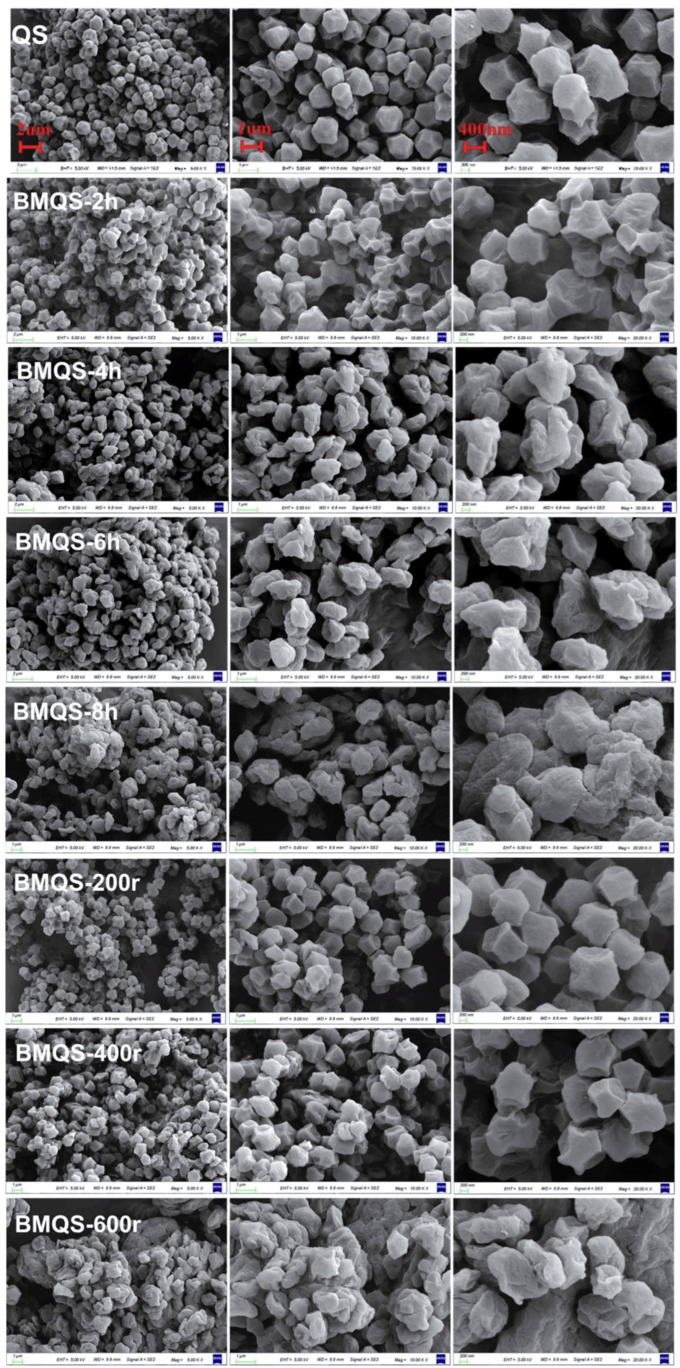
Morphology of QS and BMQS with different ball-milling times and speeds. Note: All the columns use the same scale bar.

Figure 1 shows the particle morphology of QS subjected to different ball-milling conditions. As shown in the figure, the particles of QS were typical polyhedron, and its surfaces were smooth and angular. After ball-milling, the morphology of QS particles was significantly changed, and particle fragmentation and agglomeration phenomena were appeared. A similar effect was observed for the dry ball-milled rice starch and maize starch [26,27]. During ball-milling, repeated collision, friction, and shear forces acted on the starch particles; thus, starch particles were gradually destroyed. However, with the further increases in milling time and speed, many disrupted small particles appeared; the higher surface energy of these small particles could facilitate the agglomeration of starch particles. At a shorter ball-milling time (2 h) and lower milling speed (200 r and 400 r), the particles were slightly damaged and dented due to ball-milling collision, and the surface became rough and slightly wrinkled. However, most of the particles maintained their integrity and the degree of adhesion was not serious. With the increase in milling time and speed, the polyhedral characteristics of the particles completely disappeared, the damage degree increased, and some particles stuck together to form comparatively larger particle aggregates. For ball-milled samples with a longer milling time (6 h, 8 h) and higher speed (600 r), some starch particles changed into a stripe-like formation. The intensification in the extent of aggregation with increasing milling energy input (ball-milling time and speed) might also be due to the decreased crystalline structure and increased amorphous structure. A similar reason was reported in the research of Soe et al. [28], who studied the ball-milled Thai glutinous rice starch. In the middle and late stages of ball-milling treatment, particle breakage and agglomeration occurred at the same time, it was difficult to break the dynamic balance between the crushed particles and the aggregated particles [26], and the milling efficiency decreased significantly. This was the so-called “crushing limit” effect [29,30].

#### 3.1.2. Starch Particles’ Dispersion in Water and Their Particle Size Distribution

**Figure 2:**
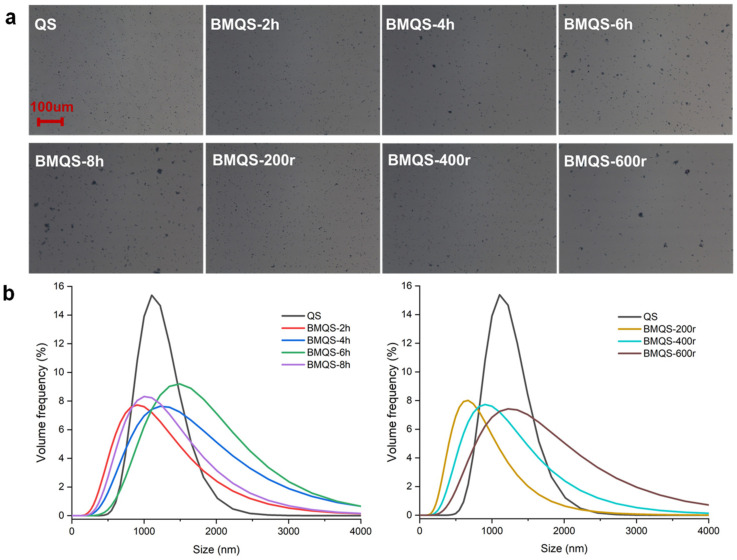
Optical images (**a**) and particle size distribution (**b**) of QS and BMQS particles with different ball-milling times and speeds, dispersed in water after homogenization. Note: All the optical pictures use the same scale bar, of 100 μm.

In order to observe the exact particle size of BMQS in the emulsion system, the particle size distribution was characterized under the same homogenization conditions (12,000 rpm, 2 min) as that of the emulsion preparation. As can be seen from Figure 2a, QS stained by iodine appeared as a dark particle when inspected under microscope. An aggregation phenomenon at a higher ball-milling time and speed were observed, which corresponded with the SEM results and the increasing volume fraction of larger particles (>2 μm) shown in Figure 2b. In Figure 2b, the particle size distribution of all the starches showed a unimodal distribution mode. The average particle sizes of QS and BMQS (2 h, 4 h, 6 h and 8 h) with increasing ball-milling times were about 1.25 µm, 1.36 µm, 1.48 µm, 1.89 µm, and 1.95 µm, respectively. The average particle sizes of QS and BMQS (200 r, 400 r, and 600 r) with different milling speeds were 1.25 µm, 0.95 µm, 1.36 µm, and 1.94 µm, respectively. A high energy input could result in a more obvious agglomeration effect [26]. All these results corresponded well with the SEM and optical images. A decreased particle size was observed for the wet-milled maize starch during milling for 25 h [14]; this different trend might be due to the combined effect of different starch types and the milling media used.

### 3.2. Supramolecular Structures

#### 3.2.1. Short-Range Ordered Structure and Double-Helix Structure

**Figure 3:**
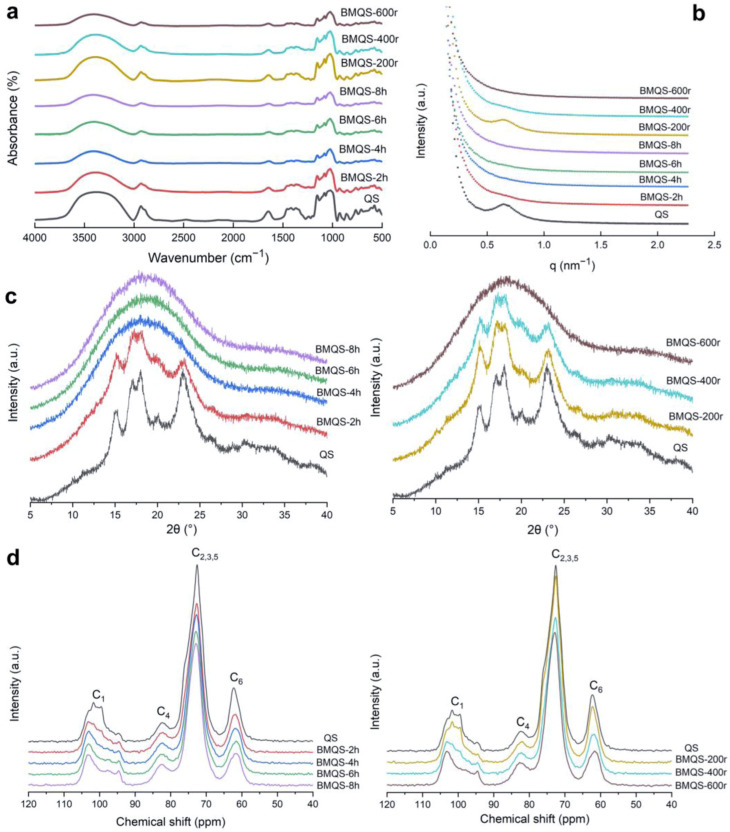
FTIR spectra (**a**), SAXS spectra (**b**), XRD spectra (**c**), and NMR spectroscopy (**d**) of QS and BMQS with different ball-milling times and speeds.

Figure 3a shows the ATR-FTIR spectra of native QS and BMQS under different ball-milling treatments. There were no missing or new characteristic peaks in the ATR-FTIR spectra of QS after ball-milling, indicating that no new functional groups were formed during ball-milling. After ball-milling, the FTIR peak position of QS did not change significantly.

In the infrared spectrum, the absorption band in the range of wavenumber 900–1300 cm^−1^ is generated by the highly coupled C=O and C-C vibrations, and this region is very sensitive to the changes in the ordered and amorphous configuration of the starch’s molecular chains, which can be used to analyze the short-range ordered structure of starch [31]. A total of 1047 cm^−1^ was found to be related to the ordered structure; 1022 cm^−1^ was found to be related to the amorphous structure. The ratio of 1047 cm^−1^ to 1022 cm^−1^ can represent the proportion of the short-range ordered structure in starch particles, and the ratio of 995 cm^−1^/1022 cm^−1^ can reflect the degree of the double helix [32]. As shown in Table 1, the value of 1047 cm^−1^/1022 cm^−1^ decreased sharply from 1.18 to 0.28 with increasing milling time (0–8 h), indicating that the short-range ordered structure of starch was seriously damaged by the increase in time. Lower decrease values were observed for the ball-milled waxy rice and waxy maize starch [33], which might be due to the different particle structures and ordered structures of these starch materials. With increasing ball-milling speed, the value of 1047 cm^−1^/1022 cm^−1^ did not significantly decrease, suggesting that the short-range ordered structure of starch was not obviously disrupted. With the increase in milling time and speed, the 995 cm^−1^/1022 cm^−1^ ratio of QS gradually increased, indicating that double-helix content increased with increasing ball-milling time and speed. These two values of ball-milled maize starch decreased [27]. It can be inferred that starch type greatly affects the impact of ball-milling on the short-range ordered structure of starch.

#### 3.2.2. Long-Range Ordered Structure

Figure 3c and Table 1 show the XRD spectra and relative crystallinity of QS and BMQS with different milling times and speeds. The X-ray diffraction pattern of QS showed five distinct diffraction peaks, at 2θ, of 15°, 17°, 18°, 20°, and 23°, which represents the typical A-type crystalline structure. With the increase in milling time and speed, the diffraction peak area and crystallinity gradually decreased, meaning there was a gradual decrease in the crystalline structure and increase in the amorphous structure [34]. When the milling time was no less than 4 h or the milling speed was no less than 600 r, the diffraction peak disappeared completely, indicating that the crystalline structure was destroyed completely under these conditions. The disappearance of the crystalline structure after ball-milling was consistent with the results of previous research [35,36,37]. The crystalline structure of starch is composed of a long-range ordered double-helix structure of the side chains of amylopectin, which are arranged in parallel by hydrogen bonds between adjacent glucose residues. The disappearance of the crystalline structure after ball-milling might be because long-range double-helix structures were no longer ordered and arranged, or due to the uncoiling of the long-range double-helix structure. However, following FTIR, the double-helix content after ball-milling greatly increased; thus, it was inferred that the disappearance of the crystalline structure after ball-milling was due to the fact that the long-range double-helix structure was no longer ordered–arranged other than the coiling of the double helix. The increase in double-helix content might be due to the rupture of the long-range double helix due to ball-milling, which caused one to break into two.

#### 3.2.3. Lamellar Structure

The lamellar structures can be tested by the SAXS instruments. The SAXS spectra of QS and BMQS are shown in Figure 3b. In general, perfect semi-crystals with a high degree of order can lead to the appearance of scattering peaks with good visibility at around 0.6 nm^−1^ [38]. The Lamellar repeat distance of starch particles can be obtained using 2π to divide the q at the peak position. The average repetition distance of semi-crystalline flakes of QS was 9.81 nm, and that of semi-crystalline flakes of BMQS-200 r was 9.94 nm, suggesting that the lamellar structure was a little looser. A similarly increased lamellar structure was observed for the ball-milled waxy maize and waxy rice starch, which was mainly due to the increased thickness of the crystalline lamellar [33]. The scattering peak of BMQS-2 h, 4 h, 6 h, and 8 h, and 400 r and 600 r disappeared, indicating that the alternating crystalline structures–amorphous structures of these starches did not exist. For BMQS-2 h (BMQS-400 r), the lamellar structure disappeared, while the crystalline structure still existed, meaning that full breakage of the amorphous lamellar and the partial disruption of the crystalline structure occurred under these conditions; thus, BMQS-2 h (BMQS-400 r) did not show an alternating crystalline structure/amorphous structure. For BMQS-4 h, 6 h, and 8 h and BMQS-600 r, the crystalline structures were fully disrupted, suggesting that both the amorphous and crystalline structures disappeared under these ball-milling conditions.

#### 3.2.4. NMR Data Analysis

Figure 3d shows the ^13^C CP/MAS NMR spectra of QS and BMQS. The peaks at 94–105 ppm are related to the C1. Triplet peaks at 99.5 ppm, 100.6 ppm, and 101.7 ppm indicate the A type crystalline structure of QS; after ball-milling (BMQS-4 h, 6 h, and 8 h, BMQS-600 r), the disappearance of these triplet peaks synchronized with the disappearance of the crystalline peaks observed in XRD. The peak position of the resonance peak at 82.4 ppm is related to C4. Both the peak at around 103 ppm, related to C1, and the peak at 82.4 ppm, related to C4, reflected the amorphous region in starch particles. The peak position at 58–65 ppm is related to C6. The position of overlapping peaks at 68–78 ppm is related to C2,3,5. Apart from the above broad peaks, the weak peak that appears at 94.7 ppm could be attributed to high-energy, twisted conformations, which are not characteristic of single helices (102–103 ppm) and double helices (99–101 ppm) [39]. The shoulder peak at around 75–76 ppm corresponds to double helices from free amylose residues. After ball-milling, the shoulder peak at 75–76 ppm disappeared, suggesting that double helices from free amylose residues were disrupted. From FTIR, it was known that the degree of double helices increased; thus, it was inferred that the increased double helix content was mainly contributed by the existing double helix of amylopectin side chains.

### 3.3. Oil Absorption Properties and Hydrophobic Properties

As shown in Table 1, the oil absorption ratio increased a little after ball-milling, which might be because, after ball-milling, the surface of the particles became rough, crystalline structures decreased, and amorphous structures gradually increased, which caused the oil to easily permeate into the starch particles and interact with the more loosened starch molecules, resulting in a slight increase in the oil absorption ratios. A positive relationship was also observed between the water absorption properties and the amorphous structures. Similarly, increased swelling properties were observed for the ball-milled rice starch, which was also believed to be connected to the increase in the disorder of its structures [26]. As seen in Table 1, all the BMQS showed a similar water content, which was lower than that of QS. After ball-milling, the contact angle of BMQS increased. With the increase in milling time and speed, the contact angle gradually increased, indicating increased hydrophobic properties. The decreased water content and increased oil absorption properties might contribute to the increased hydrophobic properties. Focusing on the structural aspect, the increased double-helix structures observed from FTIR might also benefit the increased hydrophobic properties of BMQS.

### 3.4. Emulsifying Properties

#### 3.4.1. Visual Appearance and Emulsification Index (EI)

**Figure 4:**
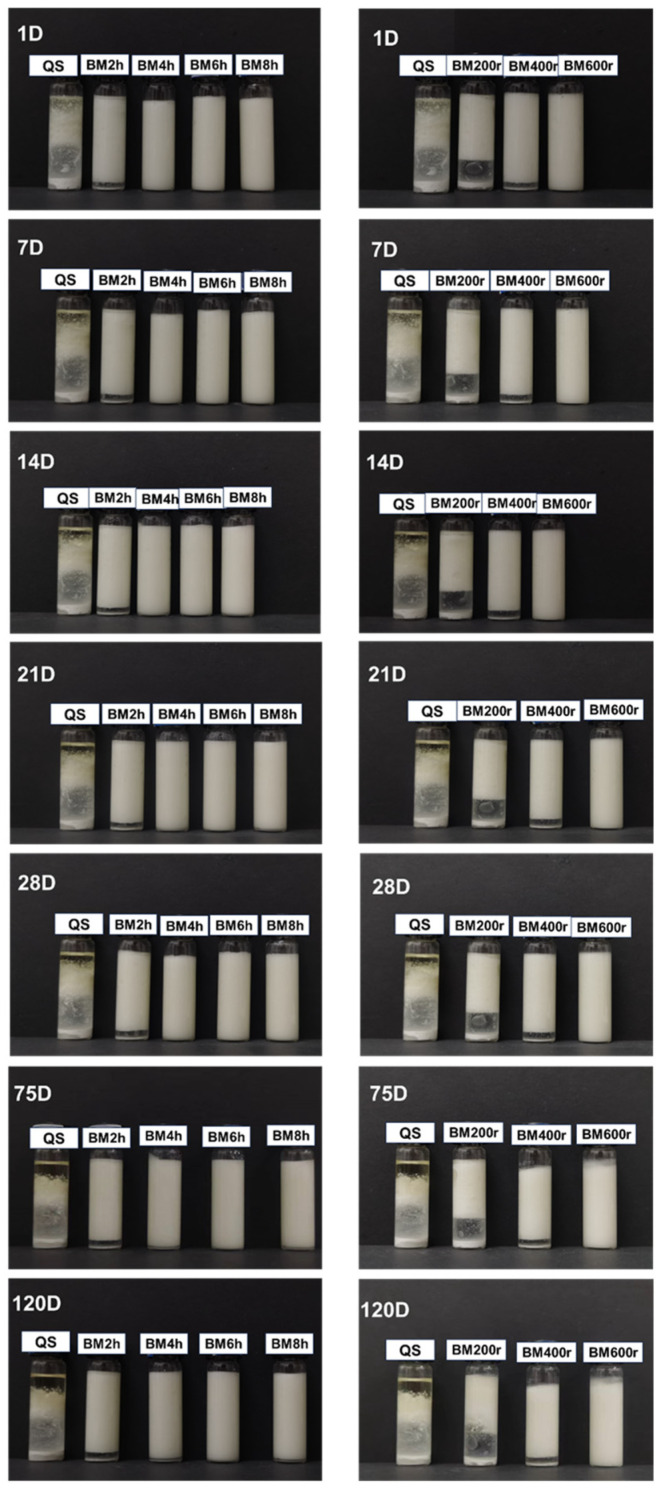
Visual diagrams of Pickering emulsions based on QS and BMQS with different milling times and speeds during 120 days’ storage.

Figure 4 and Table 2 show the appearance and emulsification index of emulsions stabilized by BMQS during 120 days’ storage. For the fresh emulsion (1 day), with the increase in milling time and speed, the emulsification index increased, suggesting that the dry milling treatment was an effective means to improve the emulsifying capacity of starch. QS showed lower emulsifying properties, which might be mainly because its higher hydrophilicity restricted its absorption at the oil–water interface. BMQS-4 h, BMQS-6 h, BMQS-8 h, and BMQS-600 r showed a full emulsification effect, suggesting that, by adjusting the milling time and speed, a full emulsification effect could be achieved. A lower milling time was needed for the dry ball-milled quinoa starch to reach a fuller emulsification state than that of the wet ball-milled maize starch [14], which might be due to the combined effect of the smaller particle size of quinoa starch and the different ball-milling method that was used. Increasing the milling speed could decrease the milling time needed for quinoa starch to reach the full emulsification state, since samples with ball-milling at 600 r for 2 h showed a similar EI to that of ball-milling at 400 r for no less than 4 h. The improved emulsifying properties might be mainly due to the increased hydrophobic properties. Moreover, focusing on the morphological aspect, with the increase in ball-milling time and speed, the appearance of some stripe-like structures could also contribute to its improved emulsifying properties. Ballard et al. [40] and Katepalli et al. [41] also stated that, compared with spherical- and polygonal-type particles, it was much easier for stripe-like starches to absorb on the oil droplets.

With the increase in storage time, the appearance of emulsions stabilized by different BMQS did not show much difference, but a small decrease in EI for some samples. After being stored for 120 days, the emulsification index of BMQS-2 h, 4 h, 6 h, and 8 h emulsions decreased by 5.98%, 3.28%, 0.91%, and 2.67%, respectively, and the emulsification index of BMQS-200 r, 400 r, and 600 r emulsions decreased by 16.30%, 5.98%, and 0%, respectively, indicating that the best stability was found for the BMQS-600 r emulsion, whose EI was still 100%, even after being stored for 120 days. For starch-particle-stabilized emulsions, part of the particles could adsorb on the oil–water interface to form a closely aligned interfacial layer to stabilize the emulsion; the other parts of the particles could fill into the void among the oil droplets, thus stabilizing the emulsion due to the steric hindrance and particle interactions [42]. The emulsion index of the BMQS-8 h emulsion decreased by 2.67% after 120 days’ storage; this decreased value was higher than that of the BMQS-6 h emulsion, indicating its relatively lower emulsifying stability, which might be due to its relatively larger starch particles. Under the same conditions, a decreased particle size was reported to be beneficial to the increased emulsifying properties [11,12]. In this research, we studied the stability of BMQS-based emulsions for 120 days, which was much longer than the most reported storage period of 2 months [13]. Most BMQS-based emulsions were stable for 120 days’ storage, indicating their better emulsifying stability.

#### 3.4.2. Emulsion Micromorphology

**Figure 5:**
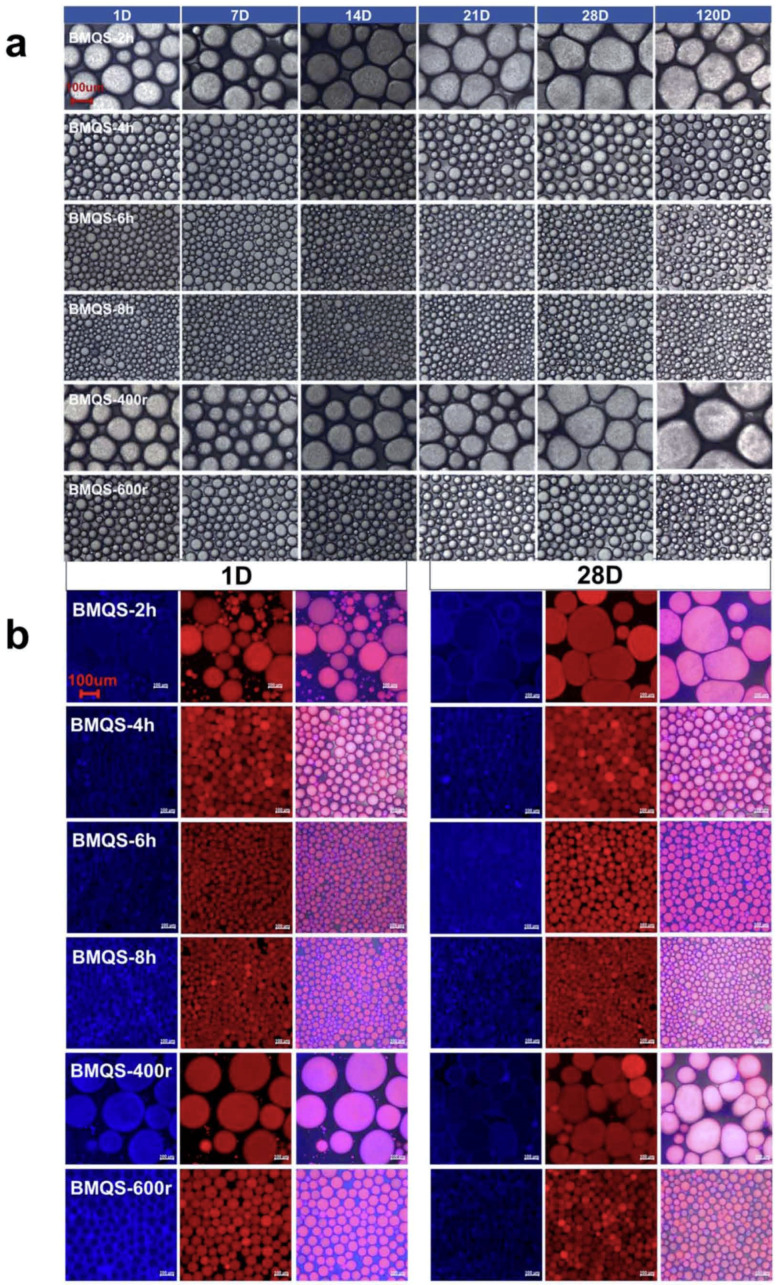
Optical microscopy pictures (**a**) and CLSM pictures (**b**) of Pickering emulsions stabilized by BMQS with different milling times and speeds during long-term storage. Left column: EMQS granules were stained with Nile blue, excited at 488 nm in blue; middle column: oil phase was stained with Nile red, excited at 633 nm in red; right column: double-excited at both 488 nm and 633 nm). Note: All the images obtained from the same testing instruments share the same scale bar.

Figure 5a shows the morphologies of the oil droplets of the BMQS-based emulsions for 120 days of storage. QS and BMQS-200 r emulsions are not shown in the figure, since only the agglomerated large oil droplets could be observed, due to their poor emulsification properties. For all the other BMQS-based emulsion systems, the oil droplets were dense and uniform. With the increase in milling time and speed, the size of the oil droplets decreased. The oil droplet sizes of BMQS-6 h, BMQS-8 h, and BMQS-600 r emulsions were smaller than that of the emulsion based on the wet ball-milled maize starch [13] and similar to that of the emulsion based on octenyl succinate starch ester [43]. This indicated that, similar to the OSA modification method, the dry ball-milling method was also a good method to improve the emulsifying properties of quinoa starch. Both the increased hydrophobic properties, the BMQS particles absorbed on the interfacial layer, and the BMQS particles appeared in the voids among the oil droplets. These could contribute to the stability of the system. The freshly prepared emulsions based on BMQS-2 h (BMQS-400 r) were large and round, and the oil droplets were arranged more evenly; after 120 days’ storage, the oil droplets became larger, the shape of the oil droplets deformed, the void between oil droplets became smaller, and the oil droplets tended to contact each other. For emulsions stabilized by BMQS-4 h, 6 h, 8 h, and 600 r, the oil droplets were evenly distributed, the interfacial layer was clearer, and the oil droplet was round and smaller; after 120 days’ storage, no obvious change was observed in the shape and size of the oil droplets for these four emulsions, indicating that these emulsions retained better stability.

#### 3.4.3. Starch Particle Distributions and Network Interactions

Figure 5b shows the CLSM images of QS- and BMQS-stabilized Pickering emulsions. The blue color represents the BMQS particles, and the red color shows oil droplets. The oil droplets decreased with the increase in milling time and speed, indicating an improved emulsification effect, which corresponded well with the optical microscope results. From the single-excited pictures, blue circles were observed surrounding the oil droplets, and a light blue color was observed on the surface of the oil droplets, which proved that part of the BMQS particles appeared as interfacial layers. In the double-excited pictures, a rose red color was observed for the oil droplets, which indirectly proved that BMQS particles were absorbed on the surface of the oil droplets; thus, the superimposed effect of the blue (BMQS) and red color (oil) resulted in the rose red color of oil droplets. Overall, some BMQS particles adsorbed on the surface of the oil droplets, acting as interfacial layers; some BMQS particles aggregated and filled in the space among the oil droplets, acting as network skeletons with oil droplets as the filling phase, facilitating the formation of three-dimensional network gels. Similar interfacial layers and network skeletons were reported in the OSA starch-based emulsions [43,44]. Both the above actions could prevent the oil droplets from gathering and improving the long-term stability of the emulsion system. As seen in Figure 5b, although emulsions with BM-2 h (BM-400 r) showed a relatively thicker interfacial layer on the oil droplets (Figure 5a,b), fewer BMQS particles filled into the space among the oil droplets, which might form weaker emulsion gel network skeletons; thus, larger oil particles with relatively lower stability were observed. Although the oil droplets of BMQS-4 h, 6 h, 8 h, and 600 r emulsion showed a relatively thinner interfacial layer, many BMQS particles filled into the space among the oil droplets, which might form stronger emulsion gel network skeletons; thus, smaller oil particles with a relatively higher stability were observed. The increased water absorption properties with increasing milling time and speed observed in Table 1 might also increase the stickiness of the starch particles and cause the swollen starch particles found among the voids of the oil droplets to interact with each other and contribute to the stronger emulsion network skeletons.

## 4. Conclusions

Dry ball-milling was used as a “cleaner-production” method to produce a “clean-label” quinoa-starch-based Pickering emulsifier with excellent emulsifying properties. Dry ball-milling significantly affected the microstructures, physical properties, and emulsifying properties of QS. With the increase in milling time and speed, the particle integrity of quinoa starch decreased, and the number of small starch particles and their agglomerates increased. With the increase in milling time and rotational speed, the short-range ordered structure, the lamellar structure, and the long-range ordered structure of starch was seriously damaged, while the double helix increased. It was much easier for the lamellar structures to be destroyed than the crystalline structure. After ball-milling, the emulsification capacity of QS was significantly improved, and the emulsions based on BM-4 h, 6 h, 8 h, and 600 r reached full emulsification state. During the 120-day storage period, the oil droplets of BMQS-2 h and BMQS-200 r deformed, the oil droplets increased, and the emulsification index decreased by 5.98% and 7.85%, respectively, while the oil droplets of BMQS-4 h, 6 h, 8 h, and 600 r emulsions did not obviously change. The BMQS-600 r emulsion had the best stability since both its oil droplets and its EI did not change after 120 days’ storage. BMQS is clean-label, low-cost, is easily and highly efficiently produced, and has great potential to be used as a Pickering emulsifier in the food, cosmetic, and pharmaceutical industries. The thermal stability of this BMQS-based emulsion should be studied further to simulate the effect of heat sterilization on this emulsion and foods based on this emulsion. Future research will focus on the application of this BMQS-based Pickering emulsifier in specific food systems.

## Figures and Tables

**Table 1:** Protein content, short-range ordered degree (1047 cm^−1^/1022 cm^−1^), double helix (995 cm^−1^/1022 cm^−1^), relative crystallinity, contact angle, water content, oil, and water absorption ratios of QS and BMQS with different ball-milling times and speeds.

Sample	Protein Content (%)	995 cm^−1^ /1022 cm^−1^	1047 cm^−1^ /1022 cm^−1^	Relative Crystallinity (%)	Contact Angle (%)	Water Content (%)	Water Absorption(%)	Oil Absorption (%)
QS	0.014 ± 0.000 ^a^	0.54 ± 0.05 ^c^	1.18 ± 0.04 ^a^	22.79 ± 0.01 ^a^	23.93 ± 2.73 ^e^	5.29 ± 0.04 ^a^	5.94 ± 0.32 ^f^	7.14 ± 0.64 ^a^
BMQS-2 h	0.013 ± 0.000 ^ab^	0.60 ± 0.07 ^bc^	1.02 ± 0.07 ^b^	9.05 ± 0.21 ^c^	35.07 ± 0.67 ^cd^	4.19 ± 0.08 ^d^	18.97 ± 0.62 ^d^	7.78 ± 0.36 ^a^
BMQS-4 h	0.011 ± 0.001 ^bc^	0.61 ± 0.04 ^bc^	0.90 ± 0.04 ^c^	-	41.17 ± 0.66 ^bc^	4.23 ± 0.03 ^d^	28.42 ± 0.88 ^c^	7.58 ± 0.32 ^a^
BMQS-6 h	0.010 ± 0.001 ^cd^	0.70 ± 0.01 ^a^	0.63 ± 0.05 ^d^	-	41.95 ± 0.45 ^bc^	4.55 ± 0.04 ^c^	31.60 ± 0.42 ^b^	7.30 ± 0.27 ^a^
BMQS-8 h	0.008 ± 0.001 ^de^	0.71 ± 0.02 ^a^	0.28 ± 0.04 ^e^	-	48.42 ± 1.13 ^ab^	4.78 ± 0.13 ^b^	33.33 ± 1.35 ^a^	7.64 ± 0.17 ^a^
BMQS-200 r	0.014 ± 0.001 ^a^	0.45 ± 0.01 ^d^	1.00 ± 0.00 ^bc^	12.42 ± 0.01 ^b^	27.68 ± 3.68 ^de^	4.70 ± 0.04 ^bc^	8.58 ± 0.43 ^e^	7.85 ± 0.11 ^a^
BMQS-400 r	0.013 ± 0.000 ^ab^	0.60 ± 0.07 ^bc^	1.02 ± 0.07 ^b^	9.05 ± 0.21 ^c^	35.07 ± 0.67 ^cd^	4.19 ± 0.08 ^d^	18.97 ± 0.62 ^d^	7.78 ± 0.36 ^a^
BMQS-600 r	0.007 ± 0.000 ^e^	0.65 ± 0.04 ^ab^	0.96 ± 0.00 ^bc^	-	50.72 ± 2.12 ^a^	4.87 ± 0.08 ^b^	33.17 ± 1.03 ^a^	7.71 ± 0.35 ^a^

Note: Data with different letters mean significant difference (*p* < 0.05).

**Table 2:** EI of Pickering emulsion based on QS and BMQS with different milling times and speeds during 120 days’ storage.

Sample	EI						
	1 day	7 days	14 days	21 days	28 days	75 days	120 days
QS	31.52% ± 8.06% ^Ad^	28.58% ± 7.05% ^Ad^	27.70% ± 7.07% ^Ad^	26.61% ± 7.07% ^Ad^	26.13% ± 7.34% ^Ad^	10.57% ± 2.71% ^Bd^	10.04% ± 2.40% ^Be^
BMQS-2 h	88.16% ± 6.16% ^Ab^	87.62% ± 6.14% ^Ab^	85.35% ± 4.66% ^Ab^	83.42% ± 5.81% ^Ab^	82.57% ± 6.79% ^Ab^	84.37% ± 2.52% ^Ab^	82.18% ± 2.81% ^Ac^
BMQS-4 h	100.00% ± 0.00% ^Aa^	100.00% ± 0.00% ^Aa^	99.16% ± 1.45% ^ABa^	98.51% ± 1.57% ^ABCa^	98.48% ± 1.37% ^ABCa^	97.18% ± 0.66% ^BCa^	96.72% ± 1.34% ^Cb^
BMQS-6 h	100.00% ± 0.00% ^Aa^	100.00% ± 0.00% ^Aa^	100.00% ± 0.00% ^Aa^	100.00% ± 0.00% ^Aa^	100.00% ± 0.00% ^Aa^	100.00% ± 0.00% ^Aa^	99.09% ± 0.31% ^Bab^
BMQS-8 h	100.00% ± 0.00% ^Aa^	100.00% ± 0.00% ^Aa^	99.38% ± 1.08% ^ABa^	99.06% ± 1.63% ^ABa^	98.78% ± 2.11% ^ABa^	97.93% ± 0.12% ^BCa^	97.33% ± 1.66% ^Cab^
BMQS-200 r	68.53% ± 1.52% ^Ac^	64.54% ± 3.32% ^Ac^	63.57% ± 4.51% ^Ac^	62.06% ± 4.12% ^ABc^	60.93% ± 6.14% ^ABc^	60.48% ± 6.26% ^ABc^	52.23% ± 1.41% ^Bd^
BMQS-400 r	88.16% ± 6.16% ^Ab^	87.62% ± 6.14% ^Ab^	85.35% ± 4.66% ^Ab^	83.42% ± 5.81% ^Ab^	82.57% ± 6.79% ^Ab^	84.37% ± 2.52% ^Ab^	82.18% ± 2.81% ^Ac^
BMQS-600 r	100.00% ± 0.00% ^Aa^	100.00% ± 0.00% ^Aa^	100.00% ± 0.00% ^Aa^	100.00% ± 0.00% ^Aa^	100.00% ± 0.00% ^Aa^	100.00% ± 0.00% ^Aa^	100.00% ± 0.00% ^Aa^

Note: Data with the different letters mean significant difference (*p* < 0.05). Different capital letters indicate the significance of the same sample in different storage periods; different lowercase letters indicate the significance of different samples during the same storage period.

## Data Availability

The datasets generated for this study are available on request to the corresponding author.
